# Impact of social media usage on academic performance of university students: Mediating role of mental health under a cross‐sectional study in Bangladesh

**DOI:** 10.1002/hsr2.1788

**Published:** 2024-01-07

**Authors:** Rana Al Mosharrafa, Taslima Akther, Fahimul Kader Siddique

**Affiliations:** ^1^ Department of Business Administration Prime University Dhaka Bangladesh; ^2^ Department of Accounting & Information Systems Jagannath University Dhaka Bangladesh; ^3^ Department of Accounting & Information Systems Begum Rokeya University Rangpur Bangladesh

**Keywords:** academic performance, Bangladesh, facebook, mental health, social media

## Abstract

**Background and Aim:**

Social media is undeniably more accessible and more appreciated today. It is undoubtedly one of the most crucial instruments for student communication. Mental health status can also meaningfully influence the students at the higher levels of the educational institutions. This study aims to evaluate the social media usage of university students and its impact on academic performance and mental health.

**Methods:**

To examine under confirmatory factor analysis (CFA) several scale measurements were confirmed by justifying the validity and reliability of several necessary indices and structural equation model. The mediation analysis was also estimated to evaluate the students' Social media addiction (employed Bergen Social Media Addiction Scale) under maximum likelihood estimation with 2000 bootstrapping and 95% bias‐corrected bootstrap confidence intervals.

**Results:**

This study shows that the usage of social media significantly improves academic performance on psychological well‐being, with a Comparative Fit Index of 0.921 and an RMSEA of 0.06 indicating a good fit of the CFA model. Finally, we exhibit a strong statistically significant positive impact of social media usage on academic success, and as supporting the hypothesis, the study observed a positive mediating role of mental health between social media addiction and academic performance.

**Conclusion:**

The present research investigations produced unique results, that is, online social media enhances mental health and mediates the link between social media addiction and academic performance in Bangladeshi students. This finding also add to the empirical database on social media usage and have significant theoretical and practical ramifications.

## 1 INTRODUCTION

Social media has become one of the most important platforms for communication in recent years. On the other side, social networking enables users to easily communicate data, files, pictures, and videos., as well as post blogs, send messages, and have in‐person chats regardless of distance. Checking and scrolling through social media have become common activities among all levels of people over the last decade. It is now easier to access the internet through the robust development of information technology. Social media dependence (SMD) is identified as a communicative reliance or behavioral intention involved in social media, driven by an uncontrolled desire to use social media, and allocating so much time and effort to social media that weakens other important life areas. The desire to express oneself on social media lights up the same part of the brain that activates while taking some addictive element.^1^ It is also found that there is an irrefutable connection between the usage of social media, bad physical and mental health, and low self‐esteem, which has become more severe due to the pandemic.^2^ Another study from California State University reported that in Bangladesh there were 45 million (27.2% of the total population) social media users in January 2021 and the number increased by 9 million (25%) between 2020 and 2021. Bangladeshi students are using different social media platforms to look for information, promote and aid communication, share e‐learning, and so forth. There have been few studies and viewpoints that have recognized four key benefits of using social media in higher education. These include strengthening connections, boosting the desire for learning, providing tailored course content, and nurturing teamwork.^3^ However, extensive use of social media is causing youths to suffer from different kinds of physical and mental disorders.

### 1.1 Literature review

Academic performance is a term used to describe a student's scholastic standings in the classroom. There is a lot of debate on the medium of measurement tool of students' scholastic ability. Though letter grade is widely accepted, it is loaded with different shortcomings. When academic achievement is measured using uniform assessments, the drawbacks can be minimized. Uniform testing would also eliminate differences in measuring performances.^4^ Grades and marks simply approximate academic performance and are therefore a vast cry from the real thing.^5^ Issues in measuring academic performance also arise when attempting to forecast future academic performance based on prior success as demonstrated by current performance.^6^ Furthermore, a lot of studies show that aspects of students' health, such as nutritional issues, physical activity, financial condition, stress level, and social support, have an impact on their academic performance and contribute to their overall grade point average.^7^ Many universities in Pakistan and other countries utilize the Grade Point Average (GPA) system as a measure of student achievement.^8^ University officials, staff members, and students must identify and improve factors that could lower barriers to reaching and maintaining the necessary GPA.^9^ Many things could prevent students from achieving and maintaining a high GPA that reflects their success in the classroom during their time at the institution. These elements could include stress, social interactions, work obligations, child care, and cognitive and learning aspects.^10^ The university staff may focus on these elements when creating ways to enhance student learning and academic success. Students in higher education are encouraged to engage in collaborative and research‐based activities through the use of some social media, including blogs and, to some extent, Wikipedia. This open access encourages participation, which can lead to chances for productive learning. Social media enables educators and learners to collaborate, publish learning‐related content (such as course materials, home assignments, test cases, and so forth), and solicit peer feedback, which is related to the principle of collaborative development among learners. The chance to adapt new ideas and modify their thinking through reflection is one of the benefits that students receive from publishing and presenting their work to a large audience through blogs, wikis, or podcasts.

Generally, addiction is defined as unchecked use of or doing something harmful. Different schools of thought expressed that individuals as rational beings who can critically judge to manage their lives. Addiction is a desire that controls one's mind and stops or from using judgment. The Impact of technology involvement has become a more and more important issue for all levels of conscious citizens. Much research has been done on it, and it has become a global concern. For example, Xue et al. (2023) stated in a study that widespread use of the internet had become a cause of mental disorders.^11^ Abuhassna et al. (2020) and Lau (2017) acknowledged that online platform use for academic concerns rarely improved academic outcomes.^12^, ^13^ Additionally, there is a negative correlation between social media usage and academic achievement, where self‐esteem possibly acts as a mediating factor.^14^

Moreover, another study also states that social media multitasking negatively predicts educational performance.^15^ According to a survey done by Raisa (2018), 41.4% of university students are highly inclined to use Facebook.^16^ Griffiths & Mamun (2019) identified that the risk of Facebook affection seems to be a crucial factor among Bangladeshi students, and depression is one of the main combined factors.^17^ There are cross‐sectional studies in Bangladesh about the association between internet obsession or problematic internet use and the mental health of school‐going adolescents.^18^, ^19^ Extreme and unrestrained social media usage has received substantial attention in recent years.^20^

Using social networks has advantages and disadvantages. It has a profound impact on how well students perform academically. The impacts of social networking use on college student's accomplishment in the classroom were examined by researchers. When the media is used excessively in a way that does not academically advance learning or its process, they discovered a terrible effect and influence.^21^ Other academics have looked at this issue, but they have come to different conclusions on whether there is a positive significant connection between students' use of social networking and their academic achievement.^22^ There is growing evidence that social media can become problematic and have several commonalities with behavioral afflictions such as gambling, gaming, loneliness, self‐esteem, depression, and so forth. However, it is also evident that the students get more enthusiastic about their wide learning environment by which they can achieve their higher academic standings and maximize their potential.

### 1.2 Study hypothesis

It's imperative to consider different perspectives while discussing how social media usage affects students. Since their level of commitment and the amount of time they spend via web‐based media might meddle with their scholastic viability and different parts of their lives. It is necessary to comprehend the causes, impacts, and way out of social media attachment before becoming concerned about it. This study seeks to ascertain the relationship between social media inclination and mental health status among Bangladeshi university students, as well as how it will expedite the students' academic performance. Hence, the following hypotheses will be proposed by this investigation.

There is a statistically positive impact of Social Media usage on Mental Health.

There is a statistically positive influence of Mental Health on Academic Performance.

Mental Health mediates positively between Social Media attachment and Academic Performance.

As, in Bangladesh, little is known about university students' entanglement with social media and how it affects mental health and academic records, the current study is trying to probe social media addiction and its effects on the mental health and academic records of Bangladeshi university students. This survey will enhance the information on social media among the students of an emerging economy.

## 2 METHODOLOGY

**Figure 1:**
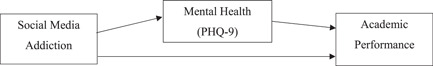
Empirical framework.

The participants of this study were some specific university students in Bangladesh. The data for this study was obtained from an online survey and offline survey administered to university students in Bangladesh between July 1 and September 30, 2022. Hence, a non‐parametric sampling technique, convenience sampling was executed in respect of accessibility within the timeframe. Overall 417 responses were collected but omitted response error and response bias, finally, 380 (91.13%) were collected for further investigation and thus, an effective response rate was achieved. The data is analyzed using AMOS V24.0 to analyze the measurements and structural relationships among the construction under investigation. Mental health is being measured by the Patient Health Questionnaire (PHQ‐9) used as a mediating variable (Figure 1) that enables tracing the depression state of an individual, respectively. The PHQ‐9 questionnaire is a nine‐item depression‐related scale that is capable of diagnosing depression, measuring the severity of the condition, and assessing the improvement of certain symptoms over time. The PHQ‐9 is a versatile tool that typically evaluates a variety of depressive symptoms, such as sadness, loss of interest in or enjoyment from activities, changes in appetite or weight, sleep problems, fatigue, feelings of guilt or worthlessness, trouble concentrating, psychomotor agitation or retardation, and thoughts of suicide or self‐harm. Due to the diversity of the student's fields of study, classes, and universities, academic performance is assessed by self‐reporting to the peers in each group. This study executed a covariance‐based structural equation model (CB‐SEM) approach due to its usefulness and applicability in executing the relationship between dependent, independent, and mediation analysis under several constructs. To do this, data screening, reliability, and validity, confirmatory factor analysis (CFA) for confronting scale measurement and validating three variables, CB‐SEM for exhibiting the effect, and mediation analysis for performing mediation analysis.

### 2.1 Variable description

For scale development, this study perceived others in follows, social media hang‐up is used in terms of the Bergen Facebook Addiction Scale, and its improved version, the Bergen Social Media Addiction Scale (BSMAS), is the most popular scale of social media inclination.^23^, ^24^ The BSMAS scale had six items, each of which was rated on a Likert scale from 1 (very seldom) to 5 (very often), with choices. The overall score was in the range of 6 to 30, and the greater the total score, the more severe the indication of social media. Secondly, The patient health questionnaire (PHQ‐9) with nine questions was evaluated as a mental health questionnaire having nine items and asked that mental health condition was estimated on tracking the state of depression symptoms over the preceding 2 weeks with responses of; 0 = “not at all,” 1 = “several days,” 2 = “more than half the days,” and 3 = “nearly every day.”^25^ The item scores are aggregated into a total score between 0 and 27 and maintained into an ordered scale of measurement that indicates the severity of depressive symptoms: 0–4 = “none,” 5–9 = “mild,” 10–14 = “moderate,” 15–19 = “moderately‐severe,” and 20–27 = “severe.” A score having 10 or above was considered as having symptoms of Major Depressive Disorder.^25^ Finally, it assessed the academic performance of the students following the recent study.^26^ Academic performance is characterized as a student's ability to complete academic activities and assess their performance across a variety of disciplines by using quantifiable metrics like final course grades and grading point averages.^27^, ^28^ The Internet become an integral part of their academic life and Facebook along with other social media are also significant components of their daily life.^29^ Access to the internet helps students get knowledge of the wider scope of resources, and improve their capabilities and skills for studies, assignments, presentations, and so forth.^30^

### 2.2 Data

The current study worked on the goal of the nexus of social media dependence and academic performance under the mediating role of mental health. To deal with this, several scales for these three pillars were assessed. Firstly, social media attachment was assessed by employing the BSMAS,^24^ Mental health was assessed by the most popular scale as it serves as a versatile tool for depression screening, diagnosis, monitoring, and severity measurement. Finally, academic performance was assessed by recent scholars.^26^ Data from this study was collected from online and printed questionnaires from different disciplines in different universities in Bangladesh. The data was collected during the COVID‐19 pandemic which started July 1, 2022 and ended on September 30, 2022. Out of 417 responses, 380 responses were considered for our investigation and 37 responses were omitted due to response error and response bias. The demographic profiles and further information were exhibited in the Appendix. In the demographic profile, out of 360 students, 228 (60%) were male and 234 (61.6%) were aged between 21 and 25 years. In the educational discipline, the Accounting information system (AIS) holds the highest possession, 150 (39.5%) whereas management 85 (22.4%), finance and banking 73 (19.2%), and the rest posit marketing and management information systems respectively.

### 2.3 Data validation and reliability

For data validation, there needs some criteria for following traditional validity and reliability for scale measurement and construct development. Firstly, internal consistency and measure of reliability are assessed under Cronbach's alpha.^31^ The threshold or acceptance criteria are different for different scholars. As Hair et al. (2010) mentioned the cut‐off level is 0.70 and a value as low as 0.60 is acceptable for exploratory research while George and Mallery (2003) mentioned 0.7 – Acceptable, but ≥0.6–Questionable.^32^, ^33^ This study observed 0.851–0.906 respectively. Construct validity (i.e., convergent validity and discriminant validity) is before CFA assessment. This validity is the ‘extent to which a set of measured items reflects the theoretical latent construct the items are designed to measure’.^34^ In convergent validity, convergent validity, two major criteria were examined‐composite reliability (CR) and average variance extracted (AVE).^35^ AVE > 0.5 and CR > 0.7 indicate adequate convergence or internal consistency.^34^ Both criteria are fulfilled in this study.

### 2.4 Confirmatory factor analysis

According to Brown (2015), CFA is a subset of structural equation modeling (SEM) that focuses on the links between observed measurements or indicators (such as test items, test results, or behavioral observation ratings) and latent variables or factors.^36^ CFA is now among the statistical techniques that are most frequently employed in practical research. This is because CFA is ideal for dealing with the kinds of queries that researchers frequently pose.

## 3 RESULT AND DISCUSSION

The perspective taken in the research result focuses on evaluating data quality through measures of reliability and validity. Internal consistency is assessed using Cronbach's alpha, with obtained values exceeding acceptable thresholds, indicating high reliability. Construct validity is confirmed through criteria like Composite Reliability and Average Variance Extracted, meeting established benchmarks, ensuring the accuracy and consistency of measurements in the study.

In Table 1, the basic statistics along with several model indices related to the measurement of scale were shown. Out of 19 variables, the average ranges between 0.55 (PHQ9: Feelings that it would be better for you to harm yourself or die) to 3.45 (BSMA6: I use social media so much that it has harmed my studies) respectively. The skewness and kurtosis of all variables did not crisscross the threshold level ‐2 to +2, hence, there is no issue of outliers or extreme values.^32^ According to normal data has kurtosis between −7 and +7 and skewness between −2 and +2. Moreover, as the sample size is greater than 200, there is no chance of impact on the Skewness and Kurtosis deviations from normality.^37^ Hence, there is no missing value issue and overall data screening is performed well.

**Table 1:** Basic statistics with study variables under three constructs.

Constructs		Mean	Median	SD	Skewness	Kurtosis	Min.	Max.
Bergen Social Media Addiction Scale (BSMAS) (α = 0.906, AVE = 0.623, CR = 0.907)	BSMAS1	3.35	3	1.135	0.148	−1.392	2	5
	BSMAS2	3.39	3	1.154	0.105	−1.437	2	5
	BSMAS3	3.42	3	1.107	0.062	−1.337	2	5
	BSMAS4	3.41	3	1.123	0.098	−1.365	2	5
	BSMAS5	3.4	3	1.157	0.111	−1.438	2	5
	BSMAS6	3.45	3	0.884	0.348	−0.641	2	5
Patient health questionnaire (PHQ‐9) (α = 0.851, AVE = 0.541, CR = 0.855)	PHQ1	0.99	1	0.721	0.734	0.987	0	3
	PHQ2	0.94	1	0.871	0.781	0.056	0	3
	PHQ3	1.18	1	0.924	0.403	−0.661	0	3
	PHQ4	1.01	1	1.045	0.523	−1.064	0	3
	PHQ5	1.1	1	0.918	0.485	−0.587	0	3
	PHQ6	0.87	1	0.884	0.918	0.24	0	3
	PHQ7	0.74	1	0.854	0.95	0.119	0	3
	PHQ8	0.76	1	0.792	0.908	0.436	0	3
	PHQ9	0.55	0	0.772	1.482	1.923	0	3
Academic performance (α = 0.858, AVE = 0.603, CR = 0.859)	AP1	2.93	3	1.301	−0.008	−1.069	1	5
	AP2	2.96	3	1.382	−0.028	−1.268	1	5
	AP3	2.96	3	1.351	0.032	−1.202	1	5
	AP4	2.93	3	1.343	0.068	−1.115	1	5

*Note*: α, AVE, and CR indicate Cronbach's alpha, average variance extracted, and composite reliability respectively.

### 3.1 Discriminant validity

Discriminant validity is measured by comparing the shared variance (squared correlation) between every pair of constructs against the average of the AVEs for these two constructs.^38^ To attain a suitable level of discriminant validity is done by examining the square root of the AVE, which should be higher than the correlations among the other variables.^35^ This study observed similar findings by following the threshold level. Additionally, another discriminant validity was studied with Heterotrait–monotrait ratio (HTMT) ratio. It estimates the true correlation of any two constructs if they are perfectly reliable and correctly measured. The HTMT criteria is a measure of the real correlation between two constructs, assuming that the measurements were accurate and trustworthy.^39^ High HTMT levels suggest that the discriminant validity may be compromised. Henseler et al. (2015) stated that an HTMT score greater than 0.90 has a serious condition of scale as it indicates a lack of discriminant validity and suggested that less than 0.85 is advised when the conceptions are theoretically more different.^39^ This study did not find any serious issues as stated in Table 2.

**Table 2:** Discriminant validity (Fornell & Larkcer discriminant validity and HTMT ratio discriminant validity).

Fornell & Larkcer discriminant validity				HTMT ratio discriminant validity			
	PHQ	BSMAS	AP		PHQ	BSMAS	AP
PHQ	0.631			PHQ			
BSMAS	0.343***	0.789		BSMAS	0.355		
AP	0.610***	0.468***	0.776	AP	0.613	0.467	

*Note*: *** indicates a strong level of statistical significance.

**Figure 2:**
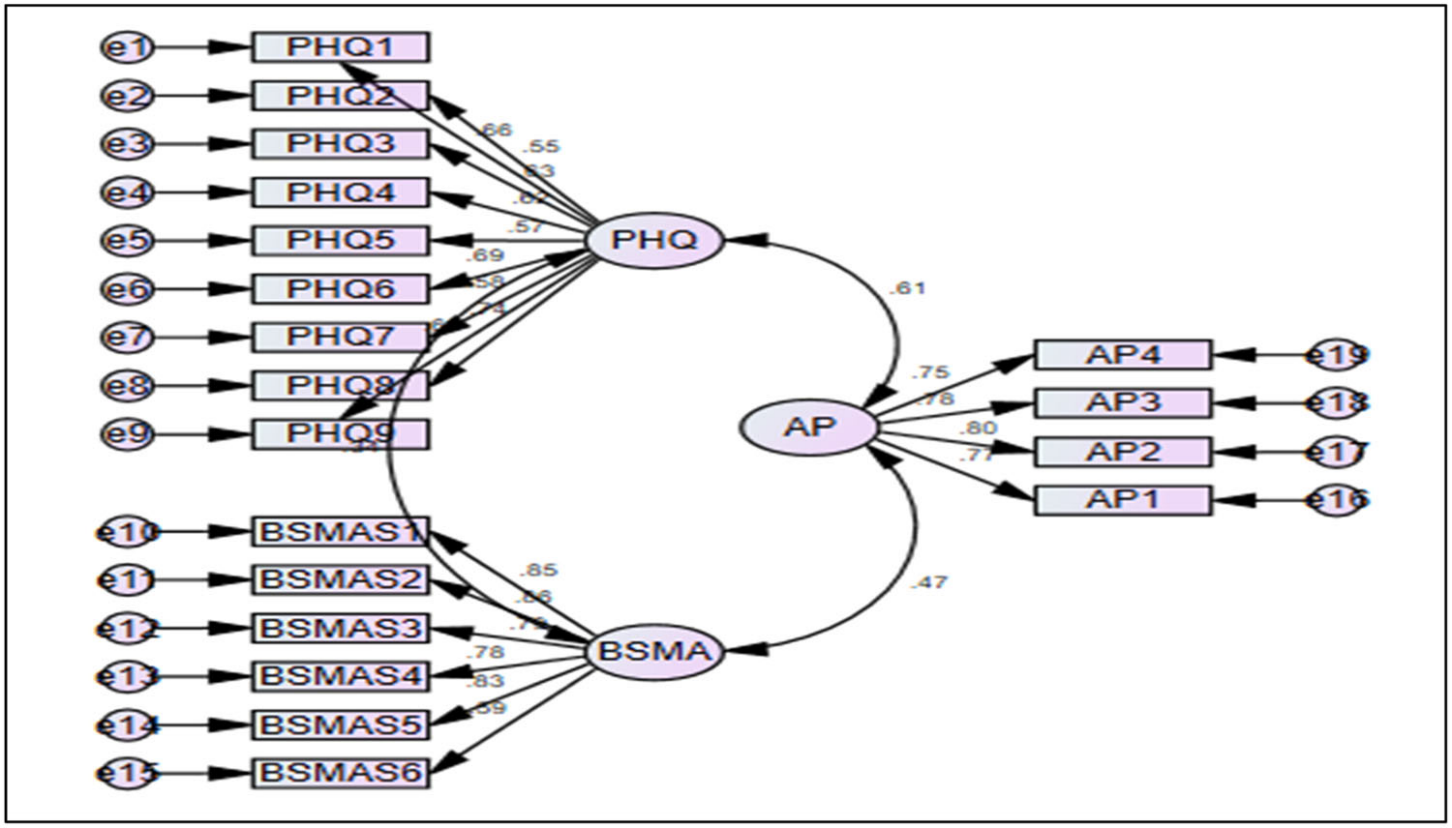
Confirmatory factor analysis of three constructs.

In the context of recent research on the development of a particular scale, CFA is used to assess factor loading. This study performed both CFA and structural equation model with maximum likelihood estimation (MLE) under 2000 bootstrapping and 95% bias‐corrected bootstrap confidence intervals considered. The factor loading represents the level of a particular construct with its regression paths. A satisfactory factor loading value is more than 0.5.^32^ For one indicator, it is deemed good when it is equal to or greater than 0.7. This threshold is followed in Figure 2. Moreover, other measurement model criteria are already discussed above.

### 3.2 Structural model and hypothesis testing

**Figure 3:**
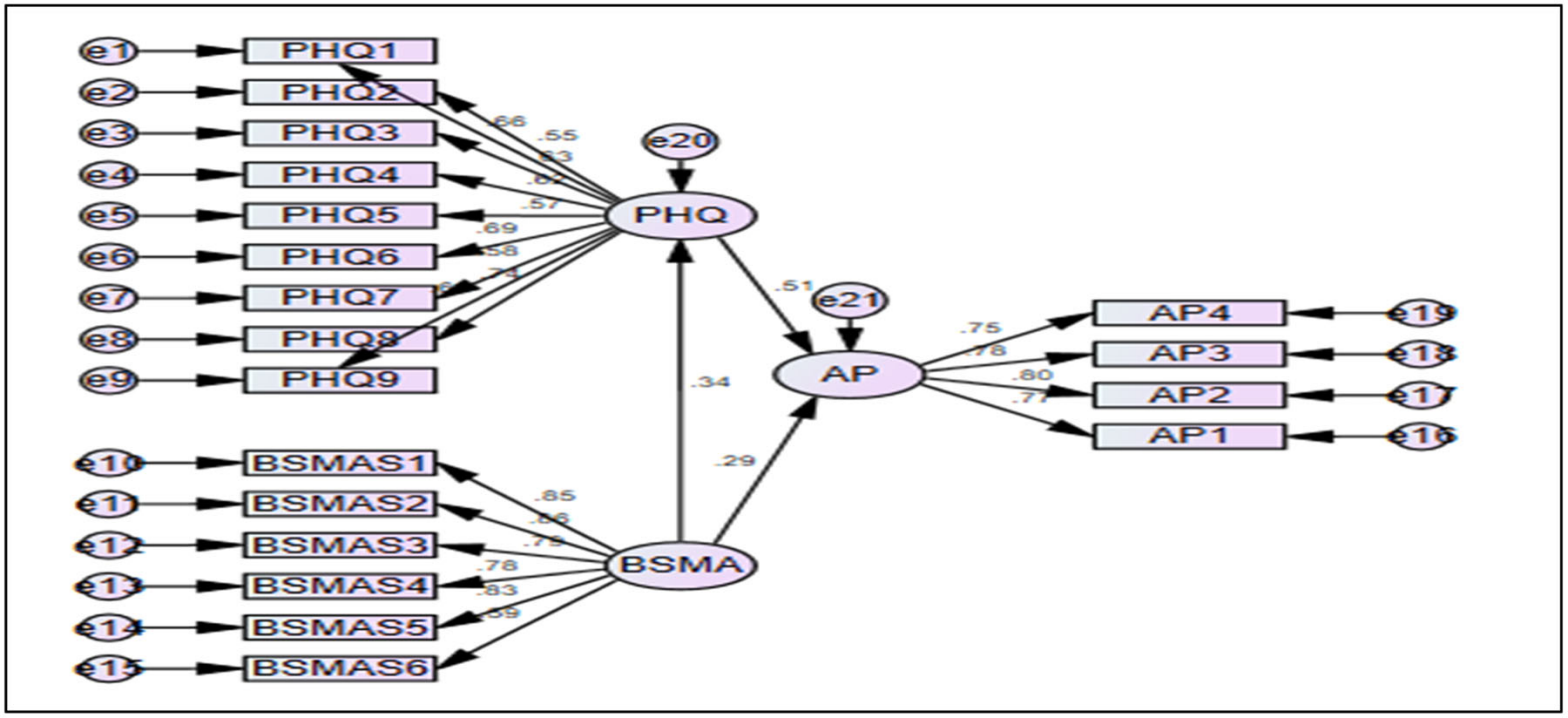
Structural equation modeling of mental health, social media attachment, and academic performance.

In the structural model, it's the pivot part of the test of the hypothesis to exhibit interrelationship among constructs.^34^ Figure 3 depicts the factor loadings with the standardized effect of the constructs. The first hypothesis was to exhibit the role of social media usage on psychological health and it came positive with a 1% level of significance (β = 0.343, t‐statistic = 4.288, *p*< 0.001). Another hypothesis was to exhibit the effect of mental health on academic performance and it also comes positive (β = 0.510, t‐statistic = 6.892, *p* < 0.001). The second hypothesis investigated the impact of social media platforms on academic performance, and it was statistically significant and had a favorable value, validating our proposition (β = 0.293, t‐statistic = 3.756, *p* < 0.001). Finally, all three hypotheses were supported and all the results stated above or below were performed on two‐sided based t‐distribution.

Using CFA, the study encountered 19 items under three constructs. The initial construct is the Bergen Social Media Addiction Scale (BSMAS), and it has 6 items. The psychological health assessment is defined in terms of PHQ‐9 having 9 items. Finally, the outcome variable was assessed on academic performance as it contains 4 items. Several fit indices were followed in case of justifying the fitness of CFA. There are three fit categories to satisfy: absolute fit, incremental fit, and parsimonious fit.^40^ According to Hu & Bentler (1995), absolute fit indices determine how well the model fits the data when covariance is observed.^41^ Three statistics were investigated under the absolute fit index: Chi‐square (2), goodness‐of‐fit (GFI), and root mean square error (RMSEA). Second, incremental fit indices coincide with two tools: the Comparative Fit Index (CFI) and the Normed Fit Index (NFI). The final tool parsimonious fit indices can be restrained by normed chi‐square (χ2/df). The following table summarizes the goodness of fit indices utilized in this research. All these criteria are illustrated in Table 3 and fulfill the cut‐off of each point.

**Table 3:** Path coefficients with model indices under structural equation model (SEM).

Path coefficients with direction				Model fitness of statistics		
Paths	Coefficients	SE	t‐statistic	Fit index	statistic	Recommended level
BSMAS → PHQ	0.343***	0.080	4.288	Chi‐square	387.738, 149 d.f.	Nonsignificant
PHQ → AP	0.510***	0.074	6.892	Chi/d.f	2.602	<3
BSMAS → AP	0.293***	0.078	3.756	RMSEA	0.06	<0.07
				GFI	0.923	>0.9
				NFI	0.901	>0.9
				CFI	0.921	>0.9
				TLI	0.931	>0.9

*Note*: *** indicates a strong level of statistical significance.

### 3.3 Mediation analysis

To explain the mediating role, we exhibit a single mediation analysis to establish a hypothesized relationship among constructs.^34^ We exhibit a single mediating effect where patient health (PHQ‐9) is a mediating variable as a third hypothesis (Table 4). Both direct and indirect effects exhibited significant output as it lies within a 95% bas‐corrected confidence interval after 5000 bootstrapping. Under the proposition of several scholars, the study observed a positive partial mediation of mental health between social media usage and academic performance.^34^ Hence, the third hypothesis was supported.

**Table 4:** Mediation analysis (test for mediation using a bootstrap analysis With a 95% Confidence Interval).

Paths	Total effect	Direct effect	Indirect effect	*p* value	Decision
BSMAS to AP via PHQ	0.510** (0.446, 0.597)	0.293*** (0.205, 0.387)	0.175** (0.128, 0.216)	0.015	Partial mediation

*Note*: **^,^*** indicates a strong level of statistical significance.

The study's limitations include potential sampling bias due to convenience sampling, self‐reported academic performance subjectivity, and the influence of the COVID‐19 pandemic on the data. Specific scales may not fully capture social media dependence and mental health complexity. Variability among students from different fields of study also impacts the findings. These limitations underscore the need for cautious interpretation and the potential for future research to address these challenges.

## 4 CONCLUSION

As university students engage in a range of social media activities daily, there are growing worries about social media's possible harmful impacts on students' well‐being. The influence of an informal attachment on a student hampers his psychosocial well‐being and sense of belonging. However, restricting the amount of time students spend on social media and scholastic attainment can improve students' potential and cognitive abilities as well as their academic achievement. Our research findings have shown that the usage of online social media has a positive impact on university students' mental health in Bangladesh and it came with significant signs. Hence, the first hypothesis was supported and the second one also does. Moreover, the mediating role of mental health positively impacts social media addiction and academic performance connection as it also came significant but in positive sign rather than negative sign. The study also provides crucial warnings about the impacts of excessive social media use on academic performance and mental health. As the epidemic has not yet been completely eradicated, social media attachment and issues with student mental health need to receive more attention. The verdict of our study highlights the favorable effects of using social platforms on students' educational achievement, including far‐reaching effects, and suggests that using social media in higher education is generally advantageous for students. Furthermore, our findings imply that policymakers and university stakeholders have to draw greater emphasis on the issues of student mental health and social media affection. Unfortunately, a substantial number of Bangladeshi university stakeholders do not offer their students mental health support services at this challenging moment.

## AUTHOR CONTRIBUTIONS

**Rana Al Mosharrafa**: Formal analysis; writing—original draft; writing—review and editing. **Taslima Akther**: Supervision; writing—review and editing. **Fahimul Kader Siddique**: Conceptualization; writing—review and editing.

## CONFLICT OF INTEREST STATEMENT

The authors declare that all authors have read and approved the final version of the manuscript. They also declare that the corresponding author has full access to all the data in this study and takes full responsibility for the integrity of the data and the accuracy of the data analysis.

## ETHICS STATEMENT

The study protocol was approved by the Research Ethics Committee, University of Asia Pacific, Dhaka, Bangladesh (UAP/REC/2021/108). The study was conducted following the principles stated in the Declaration of Helsinki. We also obtained informed electronic consent from all the participants.

## TRANSPARENCY STATEMENT

The lead author Rana Al Mosharrafa affirms that this manuscript is an honest, accurate, and transparent account of the study being reported; that no important aspects of the study have been omitted; and that any discrepancies from the study as planned (and, if relevant, registered) have been explained.

## Data Availability

The data that support the findings of this study are available from the corresponding author upon reasonable request.
